# Systematic techniques for assisting recruitment to trials (START): developing the science of recruitment

**DOI:** 10.1186/1745-6215-14-S1-O61

**Published:** 2013-11-29

**Authors:** Jo Rick, Peter Bower, David Collier, Sandra Eldridge, Jonathan Graffy, Anne Kennedy, Peter Knapp, Adwoa Hughes-Morley, Chris Salisbury, Nicola Small, David Torgerson, Shaun Treweek, Paul Wallace

**Affiliations:** 1 University of Manchester, Manchester, UK; 2 Barts and The London, London, UK; 3 Queen Mary University of London, London, UK; 4 University of Cambridge, Cambridge, UK; 5 University of Southampton, Southampton, UK; 6 University of York, York, UK; 7 University of Bristol, Bristol, UK; 8 University of Dundee, Dundee, UK; 9 University College London and NIHR PCRN, London, UK

## 

RCTs are critical to evidence-based medicine, but recruitment is routinely problematic. Robust tests of recruitment methods, where alternative approaches are compared within RCTs, are rare. The MRC funded START programme aims to test the feasibility of routinely nesting recruitment interventions in host RCTs. This will rapidly extend the evidence base, allow promising recruitment interventions to be tested across multiple trials, and improve our understanding of the impact of contextual factors on recruitment.

Newly commissioned or recruiting trials were identified via PCRN and HTA portfolios.

A total of 225 PIs were sent a flyer describing the MRC START programme with a covering email (from PCRN or HTA) inviting them to contact the team if they would like to know more about the study. Preliminary discussions were held with interested trials to screen them for suitability. Formal agreements were then put in place and trial specific intervention content was developed.

Nearly a third (32%) of PIs approached expressed an interest in the study. Just over half (52%) were excluded, reasons included incompatible recruitment strategies, timetable issues or trial size. Reaction to the additional burden placed on trials has so far been positive. Documentation has been found fit for purpose and the process of implementation supported by a ‘can do' culture and complex research skills in trial managers.

This presentation will:

• (a) describe START and report on progress to date,

• (b) discuss early findings in relation to feasibility and host trial experience,

• (c) present materials to support the recruitment of host trials.

